# Müllerian Duct Remnants on the Uterine Surface With Ovarian Serous Cystadenoma: A Case Report and Literature Review

**DOI:** 10.1155/crog/1233064

**Published:** 2026-05-30

**Authors:** Wenxin Zhong, Xiuqing Lei, Shuangyang Qin, Yun Dai, Guangming Yang, Qi Bao

**Affiliations:** ^1^ Department of Obstetrics and Gynecology, Liyang Maternity and Child Health Hospital, Changzhou City, Jiangsu Province, China; ^2^ Department of Obstetrics and Gynecology, Liyang People′s Hospital, Changzhou City, Jiangsu Province, China; ^3^ Department of Pathology, Liyang People′s Hospital, Changzhou City, Jiangsu Province, China

**Keywords:** adenomyoma, misdiagnosis, Müllerian duct remnants, pelvic mass, uterine fibroids

## Abstract

This report describes a case of a patient diagnosed with a residual Müllerian duct mass on the uterine surface at our hospital. Through analysis of this case and a review of the literature, we are aimed at reducing the risk of misdiagnosis and optimising treatment protocols for uterine cystic masses.

## 1. Introduction

Cystic uterine masses are uncommon [1], with differential diagnoses including noncommunicating functional uterine remnants (cornua uteri), degenerative myomas, adenomyosis and intramural uterine cysts [2]. Their true origin only becomes apparent during surgery [1]. We report the case of a 48‐year‐old female patient initially diagnosed with pelvic and cervical cysts. Further diagnostic evaluation revealed the mass to be multiple Müllerian remnants on the uterine surface, accompanied by an ovarian serous cystadenoma. This case underscores the importance of comprehensive diagnostic evaluation and explores the role of targeted surgical intervention in managing complex pelvic masses, highlighting the critical importance of accurate diagnosis for implementing effective treatment strategies. Through this case, we are aimed at enhancing clinicians′ diagnostic awareness and management capabilities regarding pelvic masses and their surrounding regions.

## 2. Case Report

**Figure 1:**
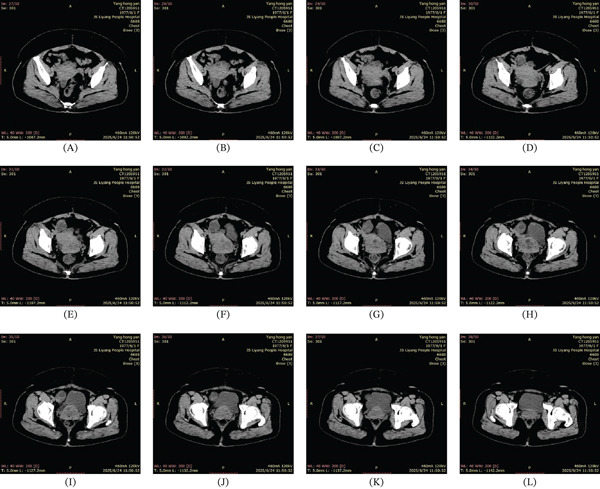
(A–L) Preoperative computed tomography (CT) features of a Müllerian duct remnants on the uterine surface with ovarian serous cystadenoma.

**Figure 2:**
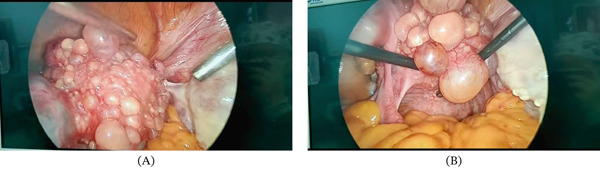
Intraoperative images of the uterus and bilateral ovaries and fallopian tubes. (A) Image of the surface of the uterus during surgery. (B) Image of the posterior surface of the uterus during surgery.

A 48‐year‐old female patient presented with irregular menstruation over the past 4 years, having sought medical attention for 1 year. An ultrasound examination at another hospital 1 year prior revealed uterine fibroids. An ultrasound performed 5 days ago showed a full‐appearing cervix with a cystic mass measuring approximately 55 × 53 × 40 mm, composed of multiple fused components. The uterine body contained several hypoechoic masses, the largest measuring approximately 12 × 9 mm. The right adnexal region contained an anechoic area measuring 36 × 35 mm, and the left adnexal region contained an anechoic area measuring 30 × 22 mm. The patient has a history of hypertension and diabetes mellitus, both managed satisfactorily with oral medication. Previous abdominal surgery includes cholecystectomy and caesarean section. She has one son and one daughter. Preoperative computed tomography (CT) was performed (Figure 1). Serum tumour marker levels for CA125, CA19‐9, AFP, CEA and HE4 were all within normal ranges. Other blood test results, including complete blood count, liver and kidney function, and coagulation parameters, showed no abnormalities. On admission, the provisional diagnosis was ‘pelvic cyst, uterine myoma’. Given the low position of the pelvic mass, a cervical cyst was considered. Following exclusion of surgical contraindications, the patient underwent exploratory laparoscopic surgery under general anaesthesia. Intraoperative findings revealed omental adhesions to the abdominal wall, an enlarged uterus with no normal tissue visible on its surface, and an irregularly shaped mass approximately 6 cm in diameter. This mass was densely packed with cystic nodules ranging from 5 to 30 mm in diameter, covering the anterior and posterior uterine walls. Cystic lesions were also observed in the left round ligament and Douglas′ pouch. The right ovary exhibited irregular enlargement with a diameter of approximately 5 cm, containing multiple cysts with the largest measuring about 35 cm in diameter. No significant normal ovarian tissue was discernible. The right fallopian tube and left adnexa showed no obvious morphological abnormalities (Figure 2). We resected the entire uterus, right adnexa and left fallopian tube.

**Figure 3:**
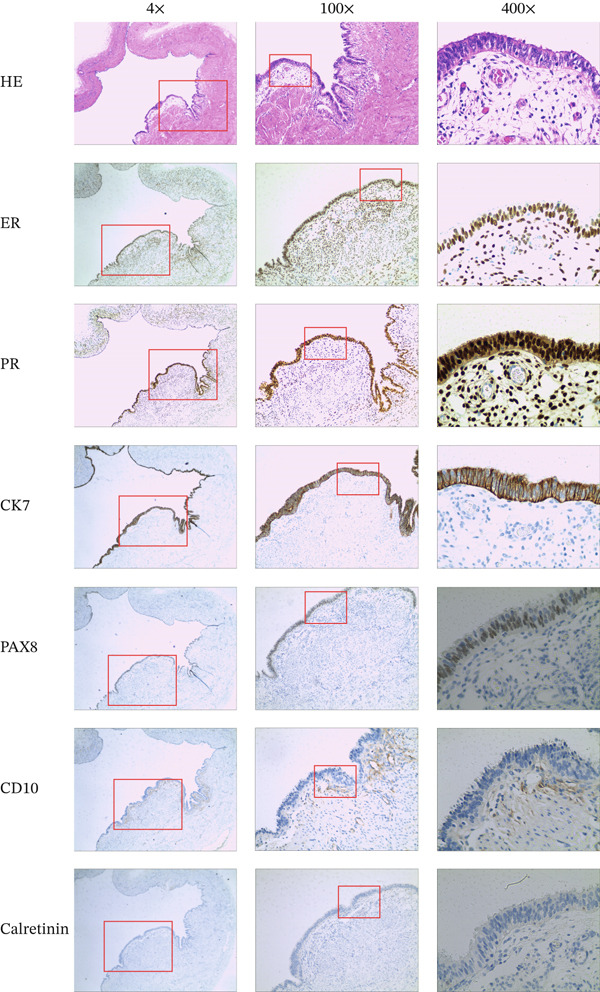
Histological examination and immunohistochemical analysis of surface tissue from the uterus.

**Figure 4:**
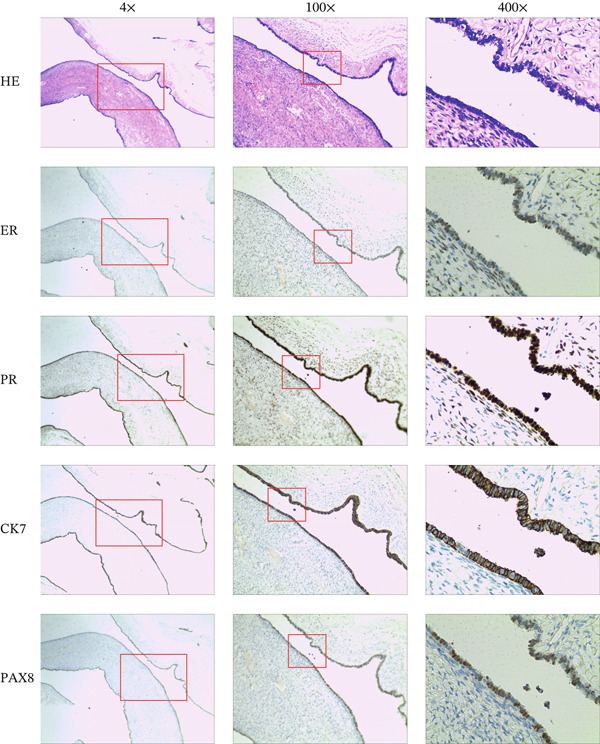
Histological examination and immunohistochemical staining of a right ovarian cyst.

The specimen was sent for rapid pathology, which reported (uterine surface) serous tumour with mild dysplasia in some glandular epithelium; further confirmation is pending routine extensive sampling. Routine pathology revealed the following: (1) (uterine surface) Based on E‐staining and immunohistochemical markers, this case is consistent with Müllerian remnant (Figure 3); (2) proliferative phase endometrium: chronic cervicitis with retention cyst formation, mild dysplasia in some glands; (3) (right adnexa) serous cystadenoma (Figure 4), dimensions: 4.0 × 3.0 × 2.0 cm; chronic inflammation of the tubal mucosa; and (4) (left fallopian tube) chronic inflammation of the tubal mucosa with one cystic adnexal mass, dimensions: diameter 0.5 cm. Immunohistochemistry results were as follows: oestrogen receptor (ER) (80%, 3+), progesterone receptor (PR) (90%, 3+), CK7 (3+), Pax‐8 (+), CD10 (partially positive), WT‐1 (+), calretinin (−), D2‐40 (−), Ki‐67 (< 5%), P53 (wild‐type), P16 (partially positive), and inhibin‐a (−). The patient recovered well postoperatively and remained recurrence‐free at 1‐month follow‐up.

This case report has obtained the patient′s verbal informed consent. All procedures were conducted in accordance with ethical standards established by institutional and/or national research committees, as well as the principles outlined in the Declaration of Helsinki as amended in 2013. This study has received approval from the Ethics Committee of Liyang People′s Hospital. The reporting of this study complies with the CARE guidelines.

## 3. Discussion

The female reproductive tract in vertebrates has undergone considerable diversification during evolution, physiologically adapting to diverse reproductive strategies [3]. Little is known about how the evolution of this organ system is driven at the molecular level. In most vertebrates, the female reproductive tract develops from paired embryonic ducts (Müllerian ducts). Postdevelopmentally, it forms the following structures: (1) the oviducts, uterus cervix, and the upper two‐thirds of the vagina and (2) the ovaries and the lower third of the vagina, which have distinct embryological origins, deriving, respectively, from germ cells migrating from the primitive yolk sac and the vaginal bulb. Normal Müllerian development relies upon the completion of three phases: organogenesis, fusion and septal resorption. The organogenesis phase is characterised by the formation of bilateral Müllerian ducts. Failure at this stage results in uterine agenesis/hypoplasia or unicornuate uterus. The fusion phase is marked by the fusion of the fallopian tubes to form the uterus. Failure here leads to bicornuate or didelphys uterus. The septal resorption phase involves the absorption of the central septum following fallopian tube fusion. Defects at this stage cause uterine septum or arcuate uterus [4]. The formation of the Müllerian duct represents a conserved process involving the shared selection of genes and molecular pathways engaged in the development of adjacent mesonephric and Wolffian duct tubules. The plasticity of Hox gene expression and Wnt signalling may underpin mammalian uterine morphological variation and vaginal evolution. This developmental plasticity in Hox and Wnt activity may also apply to other vertebrates, thereby generating the considerable morphological diversity observed in the female reproductive tract today [3].

Müllerian duct anomalies (MDA) represent a relatively uncommon phenomenon affecting adolescents and adults, as they may lead to specific gynaecological, fertility and obstetric issues. The precise incidence of MDA is difficult to ascertain [5]. These rare cystic uterine malformations associated with a normal uterus and genital tract have been described as ‘adolescent cystic adenomyosis’, ‘uterine‐like masses’ and ‘accessory lacunar uterine masses’ [6]. Müllerian duct remnants (MDR) (also termed prostatic oval cysts and Müllerian cysts) [7].

Residual Müllerian ducts have been reported in patients with the following genetic profiles: complete androgen insensitivity syndrome (46, XY karyotype and female phenotype, presenting with primary amenorrhoea at varying stages) [8–11]. Additionally, persistent Müllerian duct syndrome (PMDS) may manifest as transverse testicular ectopia [12–14], novel genetic neoplasms [15–17], cryptorchidism [18–20], incarcerated inguinal hernia [18, 21–24] or occult abdominal testicular seminoma [20]. These clinical presentations may occur singly or in combination, and not all patients exhibit associated genital hypoplasia [25].

In females, MDR may manifest as reports of ‘ectopic lumbosacral uterine masses originating from Müllerian muscle tissue, causing spinal cord tethering’ [26], inguinal uterine hernias, and Mayer–Rokitansky–Kuster–Hauser (MRKH) syndrome [27–29] (a congenital disorder characterised by hypoplastic vagina with or without concomitant uterine and/or cervical hypoplasia), potentially resulting in infertility. Approximately 200 cases of PMDS have been reported over the last 50 years, and most authors suggest leaving the Müllerian remnant in situ because of the difficulty in dissection and the presumed absence of risk of malignancy [30].

Hysterosalpingography (HSG) assesses uterine cavity and tubal patency but fails to clearly delineate the uterine contour. Ultrasound examination is rapid, readily accessible, economical and radiation‐free. However, image quality may deteriorate in larger patients or when obscured by bowel gas, and the external contour may be difficult to visualise. Three‐dimensional (3D) ultrasound represents a novel technique which, in the hands of an experienced operator, offers superior accuracy to two‐dimensional ultrasound and is comparable with or superior to MRI in assessing MDA [31]. The 3D ultrasound holds promise as the imaging standard for MDA [31]. Although pelvic ultrasound is typically the preferred initial imaging modality, pelvic MRI provides crucial additional detail [32]. MRI is currently regarded as the optimal imaging modality for MDA assessment. It is radiation‐free and clearly delineates both the internal and external uterine anatomy. MRI has demonstrated excellent concordance with the clinical diagnosis of MDA subtypes [33]. In this case, preoperative ultrasound revealed anechoic left adnexa, multiple hypoechoic areas in the uterine body and a cystic mass in the cervix. However, laparoscopic exploration showed no significant abnormalities in the left adnexa, and postoperative pathology did not identify uterine fibroids. Furthermore, the reliance solely on CT without MRI led to inadequate preoperative diagnosis and assessment.

Residual Müllerian ducts frequently lack specific clinical manifestations and are often encountered as surprising findings during other surgical procedures. Typically, surgeons face a dilemma regarding the management of MDR, namely the fallopian tubes, uterus and proximal vagina. Until recently, preservation of these structures was recommended when complete excision was considered likely to compromise their blood supply and integrity. Recent reports of malignancies arising within these preserved structures justify a more aggressive approach: excision of the mucosa from retained Müllerian structures without compromising integrity or vascularity, thereby reducing the incidence of malignancy [34]. The breadth and complexity of Müllerian anomalies have been further highlighted through the multiyear project of the American Society for Reproductive Medicine (ASRM) Müllerian Anomaly Working Committee. Collaboration among specialists in reproductive medicine and surgery, radiology and education has yielded an updated ASRM classification system for Müllerian anomalies, termed ‘ASRM MAC 2021’ [35]. A novel feature of MAC 2021 is its capacity for editing, adding and supplementing classification content. It is anticipated that future iterations will incorporate additional rare and complex anomalies into this taxonomy.

Women experiencing severe progressive dysmenorrhoea and menstrual alterations unresponsive to pharmacological treatment require specialist assessment and detailed pelvic imaging. Should uterine myometrial abnormalities be identified, accessory cavitated uterine mass (ACUM) should be included within the differential diagnosis. Standardising terminology to “ACUM” will facilitate the accumulation of additional case reports [36]. A deeper understanding of ACUM, encompassing its clinical presentation, diagnosis and management, will enhance recognition and awareness of this rare uterine anomaly.

The ER and PR serve as markers for Müllerian differentiation [37, 38]. PAX8 is a nuclear transcription factor exhibiting limited expression in both normal and neoplastic tissues in a cell‐dependent manner. PAX8 has been detected in embryonic Müllerian ducts, human fallopian tubes, and ovarian carcinomas [39]. However, little is known regarding its expression in other regions of the female reproductive tract [40]. CK7 positivity correlates with urothelial origin or mucinous ovarian carcinoma. CK7 overexpression is observed in high‐grade serous ovarian carcinomas and gastric cancers [41]. CD10 is positive in 97.9% (46/47) of genital endometriotic lesions, specifically in endometriotic stromal cells. Even in CD10‐negative cases, PAX8 demonstrates strong glandular positivity [42]. Calretinin was initially described for diagnosing mesothelioma. Within the female genital tract, calretinin was first reported in Wolffian remnants and associated lesions, subsequently employed to diagnose ovarian sex cord‐stromal tumours, endometrial stromal tumours with sex cord‐like differentiation, and uterine tumours mimicking ovarian sex cord tumours [43]. In this case, the patient demonstrated 80% strong positivity for ER (+++), strong positivity for CK7 (+++), positivity for PAX8 (+) and partial zone positivity for CD10 (+), confirming Müllerian tract presence.

Ovarian serous tumours have traditionally been considered to originate from the surface epithelium of the fallopian tubes or ovaries [44]. Previous reports suggest a potential third pathway involving mesenchymal–epithelial transition and Müllerian metaplasia [45]. Immunohistochemical confirmation establishes that the ovarian serous cystadenoma in this case also derives from the Müllerian duct.

## 4. Conclusion

This case report underscores the importance of considering the origin of uterine masses when diagnostic findings remain inconclusive. In clinical practice, careful consideration should be given to the differential diagnosis and treatment planning for both benign and malignant uterine masses.

## Author Contributions

Wenxin Zhong and Qi Bao authored the paper. Yun Dai and Guangming Yang conducted pathological analyses. Wenxin Zhong, Qi Bao, Shuangyang Qin and Xiuqing Lei conducted radiological and performed the surgical procedures.

## Funding

This study was supported by the Liyang People′s Hospital (LRY‐XKGG‐2025003).

## Disclosure

All authors have reviewed and approved the final manuscript.

## Ethics Statement

This study was approved by the Ethics Committee of Liyang People′s Hospital. The ethical code is 2025057(5 September 2025).

## Consent

This study utilised retrospective data and, as patient informed consent was waived, has received ethical approval.

## Conflicts of Interest

The authors declare no conflicts of interest.

## Data Availability

Data are available upon request due to privacy/ethical restrictions.

## References

[1] Protopapas A. , Milingos S. , Markaki S. , Loutradis D. , Haidopoulos D. , Sotiropoulou M. , and Antsaklis A. , Cystic Uterine Tumors, Gynecologic and Obstetric Investigation. (2008) 65, no. 4, 275–280, 10.1159/000113871, 2-s2.0-44849144626. 18216491

[2] Brosens I. , Gordts S. , Habiba M. , and Benagiano G. , Uterine Cystic Adenomyosis: A Disease of Younger Women, Journal of Pediatric and Adolescent Gynecology. (2015) 28, no. 6, 420–426, 10.1016/j.jpag.2014.05.008, 2-s2.0-84946490671, 26049940. 26049940

[3] Major A. T. , Estermann M. A. , Roly Z. Y. , and Smith C. A. , An Evo-Devo Perspective of the Female Reproductive Tract, Biology of Reproduction. (2022) 106, no. 1, 9–23, 10.1093/biolre/ioab166, 34494091. 34494091

[4] Chandler T. M. , Machan L. S. , Cooperberg P. L. , Harris A. C. , and Chang S. D. , Mullerian Duct Anomalies: From Diagnosis to Intervention, British Journal of Radiology. (2009) 82, no. 984, 1034–1042, 10.1259/bjr/99354802, 2-s2.0-71549138710, 19433480. 19433480 PMC3473390

[5] Shulman L. P. , Müllerian Anomalies, Clinical Obstetrics and Gynecology. (2008) 51, no. 2, 214–222, 10.1097/GRF.0b013e31816feba0, 2-s2.0-43249123258. 18463453

[6] Acien P. , Bataller A. , Fernandez F. , Acien M. I. , Rodriguez J. M. , and Mayol M. J. , New Cases of Accessory and Cavitated Uterine Masses (ACUM): A Significant Cause of Severe Dysmenorrhea and Recurrent Pelvic Pain in Young Women, Human Reproduction. (2012) 27, no. 3, 683–686, 10.1093/humrep/der471, 2-s2.0-84857215801, 22252088. 22252088

[7] Desautel M. G. , Stock J. , and Hanna M. K. , Mullerian Duct Remnants: Surgical Management and Fertility Issues, Journal of Urology. (1999) 162, 3 Part 2, 1008–1013, 10.1016/S0022-5347(01)68050-9, 2-s2.0-0032855078, 10458422. 10458422

[8] Ulloa-Aguirre A. , Mendez J. P. , Chavez B. , Carranza-Lira S. , Angeles A. , and Perez-Palacios G. , Incomplete Regression of Müllerian Ducts in the Androgen Insensitivity Syndrome, Fertility and Sterility. (1990) 53, no. 6, 1024–1028, 10.1016/S0015-0282(16)53579-1, 2112490. 2112490

[9] Nichols J. L. , Bieber E. J. , and Gell J. S. , Case of Sisters With Complete Androgen Insensitivity Syndrome and Discordant Müllerian Remnants, Fertility and Sterility. (2009) 91, no. 3, 932.e15–932.e18, 10.1016/j.fertnstert.2008.09.027, 2-s2.0-61349110710, 18930210. 18930210

[10] Güven A. , Dursun F. , Özkanlı S. , Güçlüer B. , and Kuru L. İ. , Complete Androgen Insensitivity Syndrome and Discordant Müllerian Remnants: Two Cases With Novel Mutation in the Androgen Receptor, Journal of Pediatric Endocrinology & Metabolism. (2013) 26, no. 9–10, 909–914, 10.1515/jpem-2013-0047, 2-s2.0-84888193020, 23729616. 23729616

[11] Chen D. L. , Guo S. , Chen Q. L. , Qiu S. J. , Xu Y. Y. , Zhang J. , Ma H. M. , and Li Y. H. , Complete Androgen Insensitivity Syndrome Coexisting With müllerian Duct Remnants: A Case Report and Literature Review, Frontiers in Pediatrics. (2024) 12, 1400319, 10.3389/fped.2024.1400319, 38895190. 38895190 PMC11183786

[12] Jaka R. C. and Shankar M. , Hernia Uterine Inguinale With Transverse Testicular Ectopia and Mixed Germ Cell Tumor, Indian Journal of Urology. (2007) 23, no. 1, 75–76, 10.4103/0970-1591.30274, 2-s2.0-33846666739, 19675770. 19675770 PMC2721504

[13] Telli O. , Gökçe M. I. , Haciyev P. , Soygür T. , and Burgu B. , Transverse Testicular Ectopia: A Rare Presentation With Persistent Müllerian Duct Syndrome, Journal of Clinical Research in Pediatric Endocrinology. (2014) 6, no. 3, 180–182, 10.4274/Jcrpe.1479, 2-s2.0-84908213127, 25241614. 25241614 PMC4293649

[14] Ismail M. S. , Fatima U. , Ismail A. , Bakhtiar M. , and Mazhar A. , An Uncommon Presentation of Persistent Mullerian Duct Syndrome: A 27-Year-Old Male With Transverse Testicular Ectopia, International Journal of Surgery Case Reports. (2024) 125, 110555, 10.1016/j.ijscr.2024.110555. 39488069 PMC11566888

[15] Kostopoulou E. , Eliades A. , Papatheodoropoulou A. , Sertedaki A. , Sinopidis X. , Tzelepi V. , Jang S. , Seo G. H. , and Chrysis D. , 46,*ΧΥ* DSD in an Adolescent With a Novel De Novo Variant of the NR5A1 Gene - Case Report and Literature Review, Hormones. (2025) 24, no. 1, 275–281, 10.1007/s42000-024-00589-0, 39048863. 39048863

[16] Gujar N. N. , Choudhari R. K. , Choudhari G. R. , Bagali N. M. , Mane H. S. , Awati J. S. , and Balachandran V. , Male Form of Persistent Mullerian Duct Syndrome Type I (Hernia Uteri Inguinalis) Presenting as an Obstructed Inguinal Hernia: A Case Report, Journal of Medical Case Reports. (2011) 5, no. 1, 10.1186/1752-1947-5-586, 2-s2.0-83655183389. PMC3259122 22185203

[17] Maloku H. , Shabani R. , Haliti N. , Shabani N. , Maxhuni Q. , and Ferizi R. , A Rare Case Report - Ovary Attached to Testicle Inside Hernia Sac, Urology Case Reports. (2021) 38, 101673, 10.1016/j.eucr.2021.101673. 33912395 PMC8066377

[18] Carvajal Busslinger M. I. , Kuhlmann B. , Kaiser G. , Inaebnit D. , and Zuppinger K. , Persistent Müllerian Duct Syndrome: A Case Report, European Journal of Pediatrics. (1993) 152, no. Supplement 2, 10.1007/BF02125445, 2-s2.0-0027357439. 8101817

[19] Al Harbi T. Z. , Azzam K. A. , Azzam A. , Amin T. , and Bakshi N. , Incidentally Discovered Persistent Müllerian Duct Syndrome in a 45-Year-Old Male Presenting With Germ Cell Tumor and Bilateral cryptorchidism, International Journal of Surgery Case Reports. (2018) 43, 41–44, 10.1016/j.ijscr.2018.02.002, 2-s2.0-85041922522, 29453163. 29453163 PMC5849814

[20] Inuganti R. V. , Bala G. S. , Kumar Y. K. , and Bharathi Y. K. , Persistent Mullerian Duct Syndrome With Testicular Seminoma: A Report of Two Cases, Indian Journal of Urology. (2011) 27, no. 3, 407–409, 10.4103/0970-1591.85451, 2-s2.0-80054047858, 22022070. 22022070 PMC3193747

[21] Pulido L. , Iwasiuk G. , Sparkuhl M. , Bui D. , and Springs H. , Persistent Mullerian Duct Syndrome Presenting in an Incarcerated Recurrent Inguinal Hernia With Hydrocele, Urology Case Reports. (2017) 12, 47–48, 10.1016/j.eucr.2017.02.007, 2-s2.0-85015408465. 28331809 PMC5358943

[22] Sherwani A. Y. , Shah A. Q. , Wani A. M. , Bashir A. C. , Bashir A. K. , Sofi F. A. , Wani A. A. , Lone W. , Sherwani A. H. , Sheikh M. R. , and Sharma R. R. , Hysterectomy in a male? A rare case report, International Journal of Surgery Case Reports. (2014) 5, no. 12, 1285–1287, 10.1016/j.ijscr.2014.10.020, 2-s2.0-84918822399, 25481861. 25481861 PMC4276263

[23] Dadheech D. , Om P. , Shridatt S. A. , Patni A. , and Verma N. , A Rare Case Report of Inguinal Hernia With Persistent Mullerian Duct and Klinefelter Syndrome, Journal of Clinical and Diagnostic Research. (2016) 10, no. 6, PD28–PD29, 10.7860/JCDR/2016/18361.8050, 2-s2.0-84974667714, 27504355. 27504355 PMC4963715

[24] Jafari R. , Javanbakht M. , and Dehghanpoor F. , Inguinal Herniation of Left Ovary, Fallopian Tube and Rudimentary Left Horn of Bicornuate Uterus Associated With Type 2 Mayer-Rakitansky-Kuster-Hauser (MRKH) Syndrome in a Teenage Girl: A Case Report and Literature Review, European Journal of Radiology Open. (2020) 7, 100215, 10.1016/j.ejro.2020.01.004, 32021881. 32021881 PMC6994826

[25] Morgan R. J. , Williams D. I. , and Pryor J. P. , Müllerian Duct Remnants in the Male, British Journal of Urology. (1979) 51, no. 6, 488–492, 10.1111/j.1464-410X.1979.tb03584.x, 2-s2.0-0018652106. 534830

[26] Sharma M. C. , Sarkar C. , Jain D. , Suri V. , Garg A. , and Vaishya S. , Uterus-Like Mass of müllerian Origin in the Lumbosacral Region Causing Cord tethering, Journal of Neurosurgery: Spine. (2007) 6, no. 1, 73–76, 10.3171/spi.2007.6.1.73, 2-s2.0-33847051319, 17233296. 17233296

[27] Nguyen B. T. , Dengler K. L. , and Saunders R. D. , Mayer-Rokitansky-Kuster-Hauser Syndrome: A Unique Case Presentation, Military Medicine. (2018) 183, no. 5–6, e266–e269, 10.1093/milmed/usx066, 2-s2.0-85047071160, 29415121. 29415121

[28] Al Omari W. , Hashimi H. , and Al Bassam M. K. , Inguinal Uterus, Fallopian Tube, and Ovary Associated With Adult Mayer-Rokitansky-Küster-Hauser Syndrome, Fertility and Sterility. (2011) 95, no. 3, 1119.e1–1119.e4, 10.1016/j.fertnstert.2010.09.065, 2-s2.0-79951955216. 21036352

[29] Dai Y. , Qin C. , Zhu L. , and Luo G. , Hernia Uterine Inguinale Associated With Mayer-Rokitansky-Küster-Hauser Syndrome: Three Case Reports and Literature Review, Medicine. (2023) 102, no. 5, e32802, 10.1097/MD.0000000000032802, 36749224. 36749224 PMC9901970

[30] Farikullah J. , Ehtisham S. , Nappo S. , Patel L. , and Hennayake S. , Persistent Müllerian Duct Syndrome: Lessons Learned From Managing a Series of Eight Patients Over a 10-Year Period and Review of Literature Regarding Malignant Risk From the Müllerian Remnants, BJU International. (2012) 110, 11 Part C, E1084–E1089, 10.1111/j.1464-410X.2012.11184.x, 2-s2.0-84873276494, 22540537. 22540537

[31] Deutch T. D. and Abuhamad A. Z. , The Role of 3-Dimensional Ultrasonography and Magnetic Resonance Imaging in the Diagnosis of Müllerian Duct Anomalies, Journal of Ultrasound in Medicine. (2008) 27, no. 3, 413–423, 10.7863/jum.2008.27.3.413, 2-s2.0-41149108178, 18314520. 18314520

[32] Robbins J. B. , Broadwell C. , Chow L. C. , Parry J. P. , and Sadowski E. A. , Müllerian Duct Anomalies: Embryological Development, Classification, and MRI Assessment, Journal of Magnetic Resonance Imaging. (2015) 41, no. 1, 1–12, 10.1002/jmri.24771, 2-s2.0-84918782202, 25288098. 25288098

[33] Mueller G. C. , Hussain H. K. , Smith Y. R. , Quint E. H. , Carlos R. C. , Johnson T. D. , and DeLancey J. O. , Müllerian Duct Anomalies: Comparison of MRI Diagnosis and Clinical Diagnosis, American Journal of Roentgenology. (2007) 189, no. 6, 1294–1302, 10.2214/AJR.07.2494, 2-s2.0-36448990059, 18029861. 18029861

[34] Manjunath B. G. , Shenoy V. G. , and Raj P. , Persistent müllerian Duct Syndrome: How to Deal With the müllerian Duct Remnants - a Review, Indian Journal of Surgery. (2010) 72, no. 1, 16–19, 10.1007/s12262-010-0003-x, 23133198. 23133198 PMC3452545

[35] Pfeifer S. M. , Attaran M. , Goldstein J. , Lindheim S. R. , Petrozza J. C. , Rackow B. W. , Siegelman E. , Troiano R. , Winter T. , Zuckerman A. , and Ramaiah S. D. , ASRM müllerian Anomalies Classification 2021, Fertility and Sterility. (2021) 116, no. 5, 1238–1252, 10.1016/j.fertnstert.2021.09.025, 34756327. 34756327

[36] Rackow B. W. , Accessory Cavitated Uterine Mass: A New müllerian Anomaly?, Fertility and Sterility. (2022) 117, no. 3, 649–650, 10.1016/j.fertnstert.2022.01.006, 35105449. 35105449

[37] Halimi S. A. , Maeda D. , Ushiku-Shinozaki A. , Goto A. , Oda K. , Osuga Y. , Fujii T. , Ushiku T. , and Fukayama M. , Comprehensive Immunohistochemical Analysis of the Gastrointestinal and Müllerian Phenotypes of 139 Ovarian Mucinous Cystadenomas, Human Pathology. (2021) 109, 21–30, 10.1016/j.humpath.2020.11.011, 33275953. 33275953

[38] Habiba M. , Heyn R. , Bianchi P. , Brosens I. , and Benagiano G. , The Development of the Human Uterus: Morphogenesis to Menarche, Human Reproduction Update. (2021) 27, no. 1, 1–26, 10.1093/humupd/dmaa036, 33395479. 33395479

[39] Chaves-Moreira D. , Morin P. J. , and Drapkin R. , Unraveling the Mysteries of PAX8 in Reproductive Tract Cancers, Cancer Research. (2021) 81, no. 4, 806–810, 10.1158/0008-5472.CAN-20-3173, 33361393. 33361393 PMC8026505

[40] Tong G. X. , Devaraj K. , Hamele-Bena D. , Yu W. M. , Turk A. , Chen X. , Wright J. D. , and Greenebaum E. , Pax8: A Marker for Carcinoma of Müllerian Origin in Serous Effusions, Diagnostic Cytopathology. (2011) 39, no. 8, 562–566, 10.1002/dc.21426, 2-s2.0-79957990905, 20607683. 20607683

[41] Dum D. , Menz A. , Völkel C. , de Wispelaere N. , Hinsch A. , Gorbokon N. , Lennartz M. , Luebke A. M. , Hube-Magg C. , Kluth M. , Fraune C. , Möller K. , Bernreuther C. , Lebok P. , Clauditz T. S. , Jacobsen F. , Sauter G. , Uhlig R. , Wilczak W. , Steurer S. , Minner S. , Marx A. H. , Simon R. , Burandt E. , and Krech T. , Cytokeratin 7 and Cytokeratin 20 Expression in Cancer: A Tissue Microarray Study on 15,424 Cancers, Experimental and Molecular Pathology. (2022) 126, 104762, 10.1016/j.yexmp.2022.104762, 35390310. 35390310

[42] Arakawa T. , Fukuda S. , Hirata T. , Neriishi K. , Wang Y. , Takeuchi A. , Saeki A. , Harada M. , Hirota Y. , Matsumoto T. , Koga K. , Wada-Hiraike O. , Kurihara M. , Fujii T. , and Osuga Y. , PAX8: A Highly Sensitive Marker for the Glands in Extragenital Endometriosis, Reproductive Sciences. (2020) 27, no. 8, 1580–1586, 10.1007/s43032-020-00186-7, 32430717. 32430717

[43] Portugal R. and Oliva E. , Calretinin Diagnostic Utility in the Female Genital Tract, Advances in Anatomic Pathology. (2009) 16, no. 2, 118–124, 10.1097/PAP.0b013e31819923ce, 2-s2.0-68649125806. 19550372

[44] Nik N. N. , Vang R. , Shih I. M. , and Kurman R. J. , Origin and Pathogenesis of Pelvic (Ovarian, Tubal, and Primary Peritoneal) Serous Carcinoma, Annual Review of Pathology: Mechanisms of Disease. (2014) 9, no. 1, 27–45, 10.1146/annurev-pathol-020712-163949, 2-s2.0-84897018477, 23937438. 23937438

[45] Dubeau L. and Drapkin R. , Coming Into Focus: The Nonovarian Origins of Ovarian Cancer, Annals of Oncology. (2013) 24, no. Supplement 8, viii28–viii35, 10.1093/annonc/mdt308, 2-s2.0-84886285788. 24131966 PMC3805308

